# A *C. elegans* model of electronic cigarette use: Physiological effects of e-liquids in nematodes

**DOI:** 10.1186/s40360-015-0030-0

**Published:** 2015-12-04

**Authors:** Daniel Panitz, Harsha Swamy, Keith Nehrke

**Affiliations:** Department of Biotechnology Engineering, ORT Braude College, Karmiel, Israel; Department of Medicine and Pharmacology & Physiology, University of Rochester Medical Center, Rochester, NY USA; Department Pharmacology & Physiology, University of Rochester Medical Center, Rochester, NY USA; Department of Medicine, University of Rochester Medical Center, 601 Elmwood Avenue, Rochester, Box 675, NY 14642 USA

**Keywords:** *C. elegans*, E-cig, Vaping, Nicotine, Toxicity

## Abstract

**Background:**

Electronic cigarettes (e-cigs) have recently become very popular particularly among the younger generation. These nicotine delivery devices are viewed as a preferable alternative to more conventional forms of tobacco use and are thought to reduce the risk of chronic obstructive pulmonary disease, the third leading cause of death worldwide. However, there is very little data available on the consequences of e-cig use, though recently nicotine-independent inflammatory responses have been reported. The genetic model organism *Caenorhabditis elegans* is a soil nematode whose cell biology is remarkably well conserved with mammals. Here, we used *C. elegans* to test the physiologic effects of e-liquids used to refill e-cigs.

**Methods:**

Larval worms were exposed from hatching onwards to low concentrations (0.2 %) of e-liquids, distilled e-liquid vapor, propylene glycol (PG), or M9 buffer as a negative control. E-liquids tested included grape, menthol, and V2 Red “classic tobacco” flavors. Nicotine (48 ppm) was tested as a second level variable. Stereotypical physiological outputs were then measured, including developmental rate, fecundity, locomotion, lifespan, and the induction of canonical stress signaling pathways.

**Results:**

A small but significant impairment of developmental rate and brood size was observed for PG and V2 Red treated worms compared to the negative control. Worms treated with e-liquids containing nicotine fared significantly worse than those that did not, but vaporization did not increase toxicity. Finally, both PG and V2 Red e-liquid induced an oxidative stress response in the absence of nicotine.

**Conclusions:**

PG exposure is sufficient to induce an oxidative stress response in nematodes, while nicotine is not. Both PG and nicotine independently influence physiologic measures of health and viability. The e-liquid flavorings did not significantly impact outcomes and there was no evidence for vaporization altering toxicity. These data suggest that the major physiologically significant component of e-liquids besides nicotine is likely the common solvent PG. We conclude that *C. elegans* are an appropriate model to rapidly assess parameters that may contribute to the basic cell biological effects of e-cigs.

## Background

The e-cig is a battery-powered, handheld nicotine delivery device. The basic design of most e-cig products consists of a battery, an atomizer, an airflow sensor and a cartridge which contains the liquid that is vaporized and inhaled [1]. When operated, current from the battery causes the metal coil in the atomizer to develop heat due to its electrical resistance, which is transferred onto the liquid to form a vapor phase. Air is inhaled through the airflow sensor, and passes through the atomizer, where it is mixed with the vapor phase of the heated liquid, before reaching the mouth of the user. This liquid is usually comprised from solvents, such as water, PG and vegetable glycerin, flavorings, additives and various concentrations of nicotine [2].

E-cigs are presented by the manufactures not only as a substitute to tobacco cigarettes, but also as a method of smoking cessation. Various studies have been conducted regarding the health effects and prevalence of e-cig use, as well the smoking cessation efficacy of e-cigs. However, decisive conclusions remain elusive and toxicological studies have found a variety of toxicants and carcinogens in the aerosols produced by e-cigs, though in significantly lower concentrations than in the mainstream smoke produced by tobacco cigarettes [3]. Controversy regarding the safety of e-cig use remains unsolved primarily due to a lack of clinical studies, though interestingly, it’s been known for some time that PG vapors can be toxic to plants grown in environmental chambers [4].

In the absence of data, e-cigs are advertised as being healthier and cleaner than tobacco cigarettes. In addition, it has been commonly claimed that e-cigs produce merely “harmless water vapor”. In light of this perception, it is not surprising that the prevalence of e-cig use in adolescents has increased substantially over the past few years [5]. In 2014, the University of Michigan’s Monitoring the Future study showed that teen use of e-cigs throughout the USA surpassed that of conventional tobacco cigarettes [6].

Despite the prevalent attitude that e-cigs represent a safe alternative to conventional cigarettes, it is unclear that they are entirely benign. A recent study found that both nicotine and the aldehyde acrolein, a component in the e-cig juices and vapors, caused losses in lung endothelial barrier function and were associated with increased oxidative stress and inflammatory responses [7]. In addition, formaldehyde-containing hemiacetals have been reported to result from vaping e-liquids [8]. Hence, there is an acute need for scientific evidence regarding the health implications of e-cig use, which will allow the establishment of proper regulation over their production and marketing.

In the present study, we approached the uncertainty surrounding e-cig use by examining the *in vivo* effects of e-cig vapors, as well as refill liquids, on the model organism *Caenorhabditis elegans.* A transparent multicellular eukaryote, *C. elegans* is one of the simplest organisms to possess a nervous system and has been widely used to study development, stress and aging (for review, see: [9]). More recently *C. elegans* have been used as a genetic model for studying environmental toxicology [10] and studies in worms have helped to reveal the genetic determinants of nicotine dependent behaviors following chronic drug exposure [11–13] and to assess the effects of cigarette smoke on innate immune responses [14]. Interestingly, this latter report which demonstrated dampening of innate immune responses and predisposition to infectivity observed in worms has been recapitulated in mammals [15, 16]. In fact, many of the advances made in worms have directly translated to mammalian physiology, highlighting the suitability of this model organism for health research.

Here, we utilized several standard, well-established assays to examine the physiological consequences of exposing *C. elegans* to refill juices or distilled vapor of e-cigs. Our results suggest that nematodes are an appropriate model to rapidly identify the most relevant factors that influence potentially adverse effects of e-cigs. We also find that PG, the solvent used to deliver nicotine in vaporized form, can induce an oxidative stress response, and the addition of nicotine to PG results in significantly impaired physiological outputs.

## Methods

### Strains

The following strains were utilized in this study: N2 Bristol (wild type) and CL2166 (P*gst-4::GFP::NLS*). Strains CL2070 (P*hsp-16.2::GFP*; *rol-6(su1006)*), ZG120 (P*nhr-57::GFP*; *unc-119(+)*), SJ4005 (P*hsp-4::HSP-4::GFP*), and SJ4058 (P*hsp-60::GFP*) were also tested but data from these strains is not included in the manuscript. All of these strains were obtained from the *Caenorhabditis* Genetics Center (CGC) at the University of Minnesota (Duluth, Minnesota, USA).

### Nematode culture

Wild type (N2 Bristol) *C. elegans* nematodes were cultured according to standard methods [17]. The nematodes were maintained at 20 °C on nematode growth medium (NGM) agar plates seeded with an auxotroph strain of *E.* coli (OP50), and were routinely propagated to prevent starvation.

Nematodes were exposed to the e-cig liquids (V2 Platinum E-Liquid, V2CIGS/VMR Products LLC., Miami, USA) via cultivation on NGM agar plates containing a 1:500 dilution of the commercially available cartridge refill solution. The composition of each solution can be found on the V2 website at http://www.v2cigs.com/templates/v2v3/Download/ingredients_V2.pdf. In general, these solutions are comprised of a solvent base PG, which is generally ~70 % of the solution, and are supplemented with nicotine. The e-liquids used here contained either 0 or 2.4 % nicotine, and hence following dilution the final concentration of PG in the plates was 0.14 % and that of nicotine was either 0 or 48 ppm. Negative unsupplemented controls contained a 1:500 dilution of M9 buffer. Propylene glycol (1, 2-Propanediol), nicotine ((−)-Nicotine hydrogen tartrate salt), and paraquat (N,N’-dimethyl-4,4’-bipyridinium dichloride) were purchased from Sigma-Aldrich (Saint-Louis, MO, USA)

### Production of vapor distillates

E-liquid vapors were produced by an iPV2S 7-70 W Box Mod (Green Leaf Technology Co., Ltd, Shenzhen, China) connected to a Nautilus Mini BVC Tank (Aspire, Shenzhen, China). A silicon tube was connected to the mouthpiece of the Nautilus Mini on one end, and the other end was placed in the lower part of a 50 ml conical tube, in which the condensation took place. The silicone tubing passed through a hole that was drilled in the lid of the conical tube, fastened to the edges of the hole with hot glue, and a layer of epoxy resin was applied on top of the dried hot glue to hold the tube in its place. The conical tube was suspended above liquid nitrogen inside a thermal container. Another tube passed through a second hole in the lid, and linked the tube to a capped 250 ml vented Erlenmeyer flask. The vent of this flask was connected through another silicone tube to the hub of a 60 ml syringe, which was used to draw in air through the vaping device. 750 μl of e-liquid or control was pipetted into the glass tank of the Nautilus Mini, and the device was activated for periods of 4 seconds with at least ten seconds between activation periods, both to simulate puffs as well as to avoid burning the wicking material within the atomizer. This was done several times to acquire a sufficient amount of the condensate. The Nautilus Mini was cleaned with water and the atomizer replaced between different treatments, and the tubes were cleaned with ethanol. The vaporized, distilled e-liquids were added to NGM plates in the same 1:500 dilution as the original e-liquids. The concentration of PG was presumed to be conserved and the concentration of nicotine was not significantly different from that in the original e-liquid, as determined by quantitative HPLC analysis (see below).

### Post-embryonic development assay

Synchronized embryos were prepared using a standard method of alkaline bleach treatment [17] and placed on seeded NGM agar plates containing the diluted e-liquids. Larval nematodes were imaged at 24 h intervals, beginning approximately 12 h following hatching and continuing for 3 days, using a PixeLINK PL-B621MU-KIT microscopy camera (PixeLINK, Ottawa, Ontario) attached to an Olympus MVX10 stereomicroscope (Olympus, Japan). The length of the nematodes was calculated using ImageJ 1.49 h software (Rasband, W.S., ImageJ, U. S. National Institutes of Health, Bethesda, Maryland, USA, http://rsb.info.nih.gov/ij/).

### Adult nematode lifespan assay

Worms were synchronized as described above using an alkaline bleach treatment to isolate embryos and cultured on e-liquid NGM agar plates for 3 days. 40 young adult nematodes were moved from each treatment plate to another plate that contained the same e-liquid supplemented with 0.1 mM FUDR (5-Fluoro-2’-deoxyuridine, F0503, Sigma-Aldrich, Saint-Louis, MO, USA). FUDR prevents the development of offspring and permits the first generation nematodes to be unambiguously identified as they age. The plates were maintained at 20 °C, with worms being moved to new, identical plates at least once to prevent starvation, and survival was assessed daily under a SMZ800 stereomicroscope (Nikon, Japan). Once per day, nematodes that didn’t actively move backward after being touched were counted as dead. Nematodes that were found dead of obvious causes unrelated to age, such as vulval rupture, were censored instead of being counted as dead.

### Brood size assay

Prior to the first eggs being laid by a synchronized population, several L4-young adult nematodes were individually moved onto new treatment plates where they were allowed to lay eggs for 24 h. The adult nematodes were then moved again onto separate plates at 24 h intervals over the course of 5 days. The plates were maintained at 20 °C, and the number of progeny was counted once they themselves had reached adulthood.

### Thrashing assay

Young adult nematodes were individually transferred into a drop of liquid M9 with a volume of ~5 μl, which was placed on top of glass cover slide, and the worms were allowed 10–15 seconds to recuperate. The slide was placed under a standard dissecting microscope and video recordings were acquired for a period of 15 seconds. A discrete thrash was defined as a full change in the lateral bending direction of the whole nematode corpus. The number of thrashes performed by the nematodes during the recorded period was counted, and divided by the length of the recording to calculate the frequency of thrashing.

### Fluorescent imaging

Images were obtained through the University of Rochester Light Microscopy Core on a FV1000 Olympus laser scanning confocal microscope with a 10x objective (Olympus, Japan) or using an epifluorescent rig consisting of a Nikon TE2000 inverted microscope (Nikon Instruments, Japan) equipped with a Cooke SensiCam cooled CCD and TILL monochromator (Till Photonics, Germany). All excitation-emission wavelengths and filters were appropriate for imaging GFP. Images were imported into ImageJ software (NIH) and average fluorescent intensities were calculated for each treatment (*n* = 10–14 worms and two experimental replicates).

### Analytical determination of nicotine concentration and statistics

Nicotine concentrations in the e-liquids and vapor distillates were obtained via HPLC (Shimadzu, Japan), as described [18] using a Phenomenex Gemini C18 column.

### Statistical analyses

A two-way ANOVA with Bonferroni post-hoc analysis was used to test significance. For lifespan analysis, the probability of survival was calculated and analyzed using Kaplan-Meier statistics. For HPLC analysis of nicotine content, a simple unpaired *t*-test was used.

## Results

### The physiologic effects of e-liquids in the model organism *C. elegans* are mainly due to PG and nicotine

**Fig. 1:**
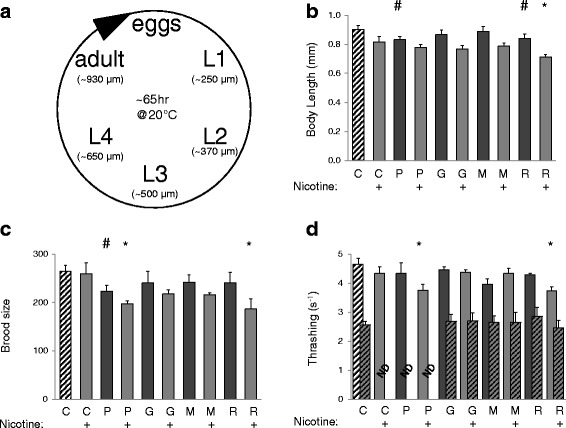
E-liquid effects on development, fecundity, and vitality in *C. elegans*. Synchronized populations of N2-Bristol strain nematode embryos were placed onto OP50 bacterial seeded NGM agar plates supplemented with PG (PG) or e-liquids containing grape (G), menthol (M) or V2 Red (R) additives, ± nicotine, as indicated. The worms were allowed to mature on these plates and physiologic effects were determined. **a** The life cycle of *C. elegans* consists of four larval stages prior to reproductive adulthood, and at 20 °C worms take ~3 days to mature. **b** Body length was assessed every day, with the ~60 h data shown. **c** Brood size was determined over the entire fertile period, starting from day 1 of adulthood (~60 h) through day 5. **d** Thrashing rates were measured using post-hoc analysis of video acquired following movement of the worms from plates to liquid M9 media, first at ~60 h and again after 7 days. Each measurement consists of at least three independent experimental replicates. The number of individual worms analyzed per replicate was from 10–20 for body size and 5 for brood size and thrashing. Two-way ANOVA with Bonferroni post-hoc analysis was used for statistical analysis. A hashtag (#) represents significant differences (*p* < 0.05) between the negative control (C) and treatments lacking nicotine, and an asterisk (*) is used to represent significant differences (*p* < 0.05) between the nicotine control (C+) and treatments containing nicotine. Though not denoted, the statistical analysis also indicated a main effect for nicotine, but no interaction between nicotine and the solvent groups. All 7 day values for thrashing differed significantly from the day 3 values but not among themselves. ND is not determined

*C. elegans* are soil nematode and widely-used as a genetic model organism. Worms develop through four larval stages prior to entering reproductive adulthood, with a normal adult measuring ~1 mm in length (Fig. 1a). The consequence of exposure to e-cig juices (e-liquids) on development was determined by supplementing Nematode Growth Media (NGM) with solvents that included 1) M9 as a negative control, 2) purified PG, which typically comprises ~70 % volume of the e-liquid, or 3) commercially available V2 Platinum e-Liquid cartridge refills. The three e-liquid flavors tested were: V2 Red (“classic tobacco”), menthol, and grape. Collectively, these five variable additives are referred to hereafter as “solvent groups”.

Exposure of worms to environmental toxicants requires their absorption from the environment, and these toxicants must pass through the cuticle. Hence, IC50 can be higher in worms than in mammals [19]. Often this necessitates an empirical determination for effective dosage. Our criteria during this process was that we choose a low enough dose of e-liquid to avoid overt changes in behavior that are known to be driven by nicotinic receptors, such as egg-laying [20], but a high enough dose to observe a slight reduction in growth rate. Our preliminary results suggested that a 1:500 dilution met these criteria. This dilution corresponds to ~0.16 % PG in the NGM plates, and for e-liquids that contain 2.4 % nicotine, which is the highest concentration available in the V2 Platinum series, this equates to 48 ppm nicotine . Reference controls were supplemented with purified PG and nicotine obtained commercially. The plates were seeded with OP50 bacteria, the normal food source fed to worms in a laboratory setting, and worms were grown from synchronized embryos. Growth rates were calculated by measuring nematode length at 24-hour intervals over 3-days, corresponding to developmental times of approximately 12, 36 and 60 h (Fig. 1a). All treatments were performed in parallel, with 10–20 worms measured per treatment and three experimental replicates performed at different times

None of e-liquids exerted overtly malign effects, and the treated worms appeared morphologically normal (data not shown). As expected, there was a main effect of nicotine (Fig. 1b), but this effect was not dependent upon the solvent group. Unexpectedly, however, PG treated worms were smaller than the negative controls, independent of nicotine (Fig. 1b). This was also true for V2 Red. These results suggested that PG and nicotine are the main contributors to the observed alterations in size. The fact that these differences were generally mirrored at earlier time points (data not shown) suggests that this was an effect of growth rate rather than a reduction in terminal size.

Growth rates in *C. elegans* often reflect dietary intake and metabolism. Another stereotypical measure of metabolism is brood size. After reaching adulthood, hermaphrodite *C. elegans* begin to lay eggs, with a fertile period extending ~3 days and a normal brood size of ~250-300 progeny. In order to support this astounding fecundity, worms need to convert their body mass into embryos on a daily basis. Most of this energy conversion occurs in the intestine, which is the site of nutrient uptake, fat storage, and vitellogenin synthesis. Hence, brood size is tightly coupled to nutrient availability and has been used as a measure of metabolic normalcy. Dietary restriction as well as stress or genetic mutations in signaling pathways that respond to stress have all been shown to influence embryo output [21–23]. Here, we assessed the effect of e-liquids on total brood size, measuring the average from five worms per treatment, with three experimental replicates performed at different times. As was the case with developmental rate, the data in Fig. 1c suggest that brood size is also reduced by exposure to PG. The p-value for the other solvent groups was suggestive of an effect, but did not reach significance. Finally, as with development, there was a main effect of nicotine, but no interaction between nicotine and the solvent groups.

*C. elegans* have also been used extensively to study aging, and many types of stress have been shown to elicit changes in lifespan through specific genetic determinants (for review, see [9]). However, in our hands two independent analyses using 20–40 worms per treatment showed that the e-liquids did not significantly influence lifespan (data not shown). In contrast to lifespan, vitality or “graceful aging” refers to the rate at which age-related physiological changes occur during an organism’s lifespan. Vitality has traditionally been correlated with locomotory rates in worms, which tend to decrease with age. Here, we used thrashing assays to assess vitality, where worms are placed in a drop of liquid M9 buffer and their rate of body bends are calculated post-hoc via recordings obtained on a stereomicroscope. Recordings were made of young adult worms and compared to older adult worms as a function of e-liquid exposure throughout life. The data in Fig. 1d represents the average of three independent replicates of five individual worms per replicate. As with the other measures tested, there was a main effect of nicotine (Fig. 1d). The worms that were exposed to PG and V2 Red containing nicotine fared significantly worse than their counterparts exposed to nicotine alone (Fig. 1d). As expected, the thrashing rate was reduced in older worms, but there was no difference among solvent groups or as a function of nicotine (Fig. 1d).

### PG and e-liquids elicit an SKN-1/Nrf-2 stress response independent of nicotine

**Fig. 2:**
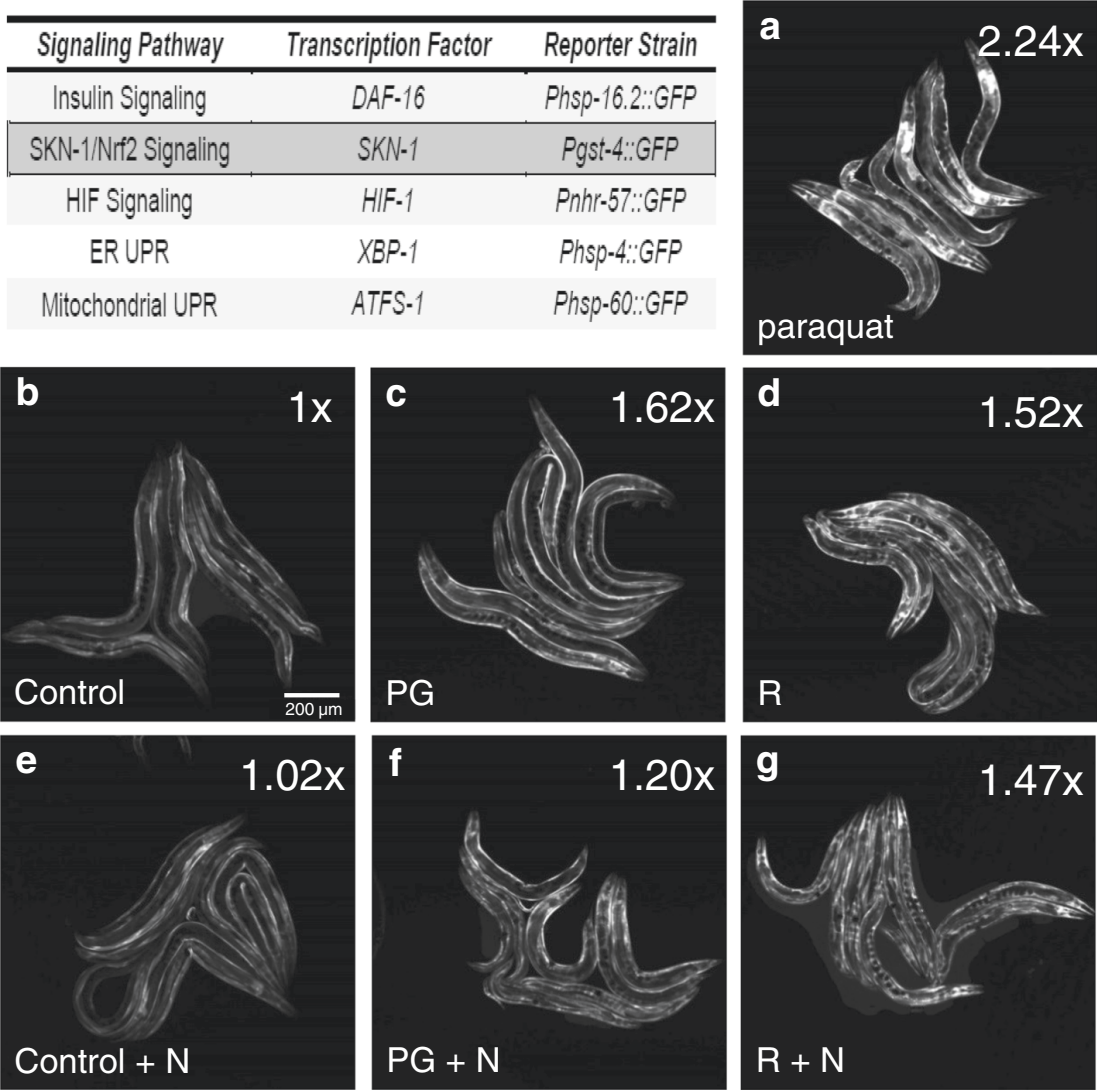
E-liquid effects on stress response pathway induction. Transgenic reporter strains containing promoter::GFP fusion proteins were used to measure induction of five canonical signaling pathways by the transcription factors shown in the table. The highlighted signaling pathway mediated by the worm Nrf-2 ortholog, the detoxification/antioxidant response factor SKN-1, is activated in response to e-liquids as shown in panels A-F. Transgenic worms expressing the SKN-1 reporter *gst-4*::GFP were grown from embryos on NGM agar plates supplemented with **a**) 10 mM paraquat as a positive control, **b**, **e**) reference controls **c**, **f**) PG (PG) or **d**, **g**) V2 Red e-liquid (R), in the **b**-**d**) absence or **e**-**g**) presence of nicotine (N), as indicated. The images shown are representative from several experimental replicates and were acquired and processed using identical settings. Average fold induction is indicated on the upper right corner of each panel. The scale bar is 200 μm

*C. elegans* stress responses are well-conserved with mammals, particularly at the cell biological level, and are represented by canonical signaling pathways that have been extensively characterized through genetic approaches. Critical transcription factors have been identified in pathways that respond to various types of stress, including nutrient availability, such the FOXO ortholog DAF-16, which is the terminal effector of the worm insulin-like signaling cascade [24]; oxidative stress, such as the Nrf-2 ortholog SKN-1 [25]; hypoxia, such as HIF-1 [26]; and proteotoxicity, such as XBP-1 [27] and ATFS-1 [28], which act at the level of endoplasmic reticular and mitochondrial unfolded protein responses, respectively. One of the advantages of working with *C. elegans* is a wealth of genetic reagents. For the purposes of this work, those reagents included transgenic strains where the promoters from specific targets of each of these transcription factors, shown in Fig. 2, have been fused to the green fluorescent protein GFP. Hence, fluorescent output can be used as a surrogate for signal pathway induction.

Each of the five transgenic strains shown in Fig. 2 was screened for induction by either PG or the V2 Red e-liquid ± nicotine using fluorescent microscopy (e-liquids containing other flavorings were not tested). Of the five strains tested, only one exhibited visible activation: the detoxification/antioxidant response factor SKN-1. Expression of the SKN-1 target gene *gst-4* was visible under basal conditions (Fig. 2b), but was greatly enhanced by exposure to 10 mM paraquat, a stereotypical oxidative stress widely used in worms (Fig. 2a). Expression was also increased, though less dramatically, in response to PG and V2 Red (Fig. 2c, d, f, and g). Induction was independent of nicotine (Fig. 2e). These data indicate that PG is sufficient to elicit a mild oxidative stress response in worms, while nicotine is not.

### Distilled vapor extracts of e-liquids exert similar effects as e-liquids themselves

**Fig. 3:**
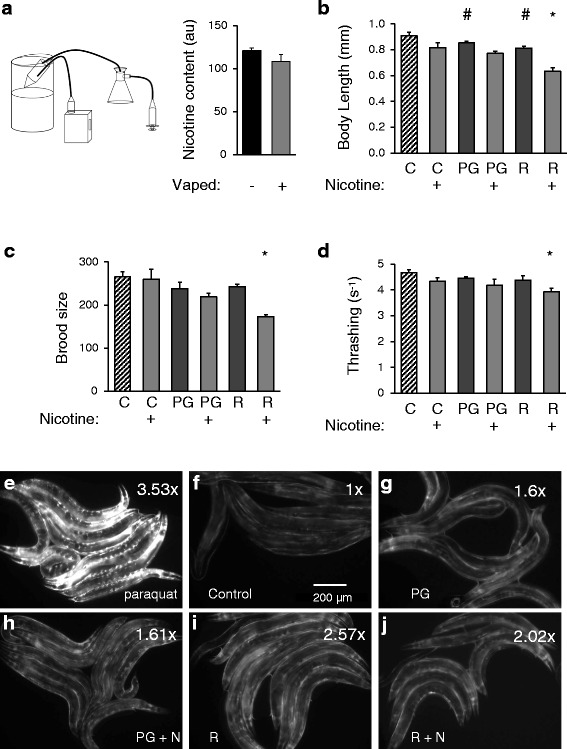
Vapor distillate effects on development, fecundity, vitality and induction of the SKN-1 stress response pathway in *C. elegans*. **a** A schematic of the system used to vaporize and collect condensates of the distilled vapor. A syringe was used to pull vapor from an activated e-cig device, using brief pulls and burn times to recapitulate what a normal user might experience during vaping. The vapor was condensed into an extract in a flask suspended over liquid nitrogen. Nicotine recovery was confirmed by quantitative HPLC analysis of the pre-vaped (−) and vaped (+) e-liquids, as indicated. Synchronized populations of N2-Bristol strain nematode embryos were placed onto OP50 bacterial seeded NGM agar plates supplemented with vaporized, distilled PG (PG) or e-liquids containing V2 Red (R) flavoring ± nicotine, as indicated. The worms were allowed to mature on these plates. **b** Body length was assessed every day, with the ~60 h data shown. **c** Brood size was determined over the entire fertile period. **d** Thrashing rates were measured using post-hoc analysis of video acquired following movement of the worms from plates to liquid M9 media. Each measurement consists of at least three independent experimental replicates. The number of individual worms analyzed per replicate was from 10–20 for body size and 5 for brood size and thrashing. The negative reference control data is replicated here from Fig. 1 for comparison. Two-way ANOVA with Bonferroni post-hoc analysis was used for statistical analysis. A hashtag (#) represents significant differences (*p* < 0.05) between the negative control (C) and treatments lacking nicotine, and an asterisk (*) is used to represent significant differences (*p* < 0.05) between the nicotine control (C+) and treatments containing nicotine. Though not denoted, the statistical analysis also indicated a main effect for nicotine, but no interaction between nicotine and the solvent groups. **e**-**j** Representative fluorescent images of transgenic worms expressing the SKN-1 reporter P*gst-4*::GFP on plates containing: **e**) 10 mM paraquat, **g**)a vapor distillate of PG (PG), **h**) a vapor distillate of PG + nicotine (N), **i**) a vapor distillate of V2 Red e-liquid (R), or **j**) a vapor distillate of R + N, as indicated. The negative control is shown in panel F. The images were acquired and processed using identical settings. Average fold induction is indicated on the upper right corner of each panel. The scale bar is 200 μm

One of the concerns regarding e-liquid vapors is that the vaporization process may result in free radical formation, which could cause oxidative stress and ultimately result in tissue damage over time. Given our observation that the detoxification/antioxidant response factor SKN-1 target gene *gst-4* is induced by e-liquids, we hypothesized that vaporization may exacerbate this responses. In order to expose worms to vaporized e-liquid, a distillation system was devised where an e-cig device was used to create a vapor that was then pulled through negative pressure into a flask suspended over liquid nitrogen (Fig. 3a). The parameters used for this distillation process mimicked those that a typical e-cig user would employ (Methods). HPLC analysis of nicotine content following distillation suggested that most of the vaporized nicotine was recovered (Fig. 3a). The distilled vapor extract was added to NGM plates at an identical dilution as the original e-liquid (1:500) and embryos were allowed to develop on these plates. While this model does not recapitulate the acute exposure to vapor experienced by an e-cig user, it did allow us to determine whether vaporization itself led to increased toxicity in the worm model. In this study, vapor extracts of PG were compared to the V2 Red e-liquid; the other flavorings menthol and grape were not tested. In addition, since e-cig devices are built to vape PG or glycerin based solvents, the negative control data, generated using an aqueous solvent, was not vaped and is replicated here from Fig. 1 strictly for comparison.

Somewhat surprisingly, most of the physiologic measurements, including body length, brood size, and thrashing rate, were similarly affected by treatment with the distilled vapor extracts as by the original e-liquids (Fig. 3b-d). However, several of these effects were suggestive but not significant. Nevertheless, there was no overt increase in toxicity following vaporization.

Finally, we observed that SKN-1 pathway induction of *gst-4*::GFP was higher in treated groups compared to controls, but was reduced compared to paraquat, suggesting that PG induces a sub-maximal stress response (Fig. 3e-j). It’s possible that PG triggers SKN-1 activity, which is essential for development of embryos and upregulates the expression of genes that result in modification, conjugation, and export of xenobiotics (for review, see [29]), in the context of detoxification rather than oxidative stress. However, we also recognize that free radicals are transient reactive molecules, and hence it’s also possible that the nature of the model, which does not permit exposure immediately upon e-liquid vaporization, may prevent us from observing their full effect.

## Discussion

The paucity of data regarding the health effects of e-cigs and vaping led us to utilize the nematode *C. elegans* as a genetic model to test the physiological and potential pathophysiological effects of exposure to e-liquid cartridge refill solution or a vaporized and condensed, distilled version of the same. The measures that were assessed reflect development, metabolism, stress responses, and vitality. The advantages of utilizing a model organism for this approach are evidenced by the scope of the study, which simultaneously interrogated the effects of the solvent PG, additives and nicotine on physiologic output and stress response pathway activation. The limitations include the absence of lung epithelia in nematodes and the corresponding lack of practical benefit to be gained by exposing them to the vapor phase. These facts may limit our ability to observe the immediate effects of ROS created during the vaping process, but do not preclude insight into the basic cell biological processes influenced by the compounds themselves.

Of the stress response pathways interrogated by fluorescent microscopy of GFP tagged target gene expression in transgenic animals, only the detoxification/antioxidant response factor SKN-1 appeared to be activated by e-liquids. While SKN-1 activation facilitates stress resistance and longevity, it has been shown that these protective effects come at the expense of growth and reproduction [30]. In general terms, nematode brood size is very plastic, with fertility sacrificed for survival under conditions of stress. Correspondingly, we found that the 3-day average body length and total brood size were reduced in groups treated with PG or V2 Red e-liquid. However, SKN-1 activation appears to be less robust in response to e-liquid treatments than paraquat (Figs. 2 and 3).We conclude that PG, the major solvent used for most e-liquids, is not benign and can itself contribute to toxicity and oxidative stress. These effects are likely mild, as canonical mitochondrial and insulin-like signaling genetic mutants that are nonetheless viable are much more severely affected [21, 23]. It is also worth noting that the data for the grape and menthol groups was suggestive, but ultimately statistical analysis did not support an effect compared to the control groups. Intriguingly, the menthol e-liquid contains 56 % PG, compared to 70 % for the other groups. Moreover, it is possible that other minor constituents of the e-liquid may contribute to this discrepancy.

In all cases, a main effect of nicotine was observed, independent of solvent group. The concentration of nicotine used was well below that which is expected to have an influence on egg-laying, a canonical behavior driven by nicotinic receptors. Our results indicate that body length, brood size, and locomotion were generally reduced when nicotine was present. However, recent studies have shown that e-liquids and vapors cause significant inflammatory and oxidative stress responses in pulmonary and airway endothelia ([31, 32]) and that vaping can lead to formaldehyde-containing hemiacetals generated from e-liquids [8]. The extent to which these effects are mediated by PG versus nicotine remains to be seen. Finally, although our data do not support a role for flavorings in altering physiologic outcomes (with the single exception of vaporized, redistilled V2 red in the context of developmental size), it is possible that other additives, such as cinnamon which has been heavily scrutinized based upon a recent report [33], may exert additional effects.

Our findings here underscore the need for more research regarding e-cigs. Adolescent use of e-cigs has only increased since their introduction to the market. E-cigs are often portrayed as benign nicotine delivery devices, encouraging people to indulge without fear of any negative consequences. Our work, along with the work of many others, may indicate that the use of these devices comes with its own risks.

## Conclusions

E-liquids and their vapor extracts cause an increase in the detoxification/antioxidant response factor SKN-1 activation, indicative of oxidative stress, and lead to impaired growth, reproduction, and vitality. PG is the main effector that induces an oxidative stress response, while both PG and nicotine independently contribute to the physiologic effects of e-liquids in worms. Our results highlight the usefulness of *C. elegans* as a model organism for interrogating how specific aspects of vaping such as flavorings, nicotine content, or even the e-cig wattage may interact with genetic determinants to induce toxicity.

## Abbreviations

PG: Propylene glycol
e-cig: Electronic cigarette
NGM: Nematode growth medium
HPLC: High performance liquid chromatography
